# Recent Advances in Bio-Based Additive Flame Retardants for Thermosetting Resins

**DOI:** 10.3390/ijerph19084828

**Published:** 2022-04-15

**Authors:** Adriana Dowbysz, Mariola Samsonowicz, Bożena Kukfisz

**Affiliations:** 1 Department of Chemistry, Biology and Biotechnology, Bialystok University of Technology, Wiejska 45A Street, 15-351 Bialystok, Poland; m.samsonowicz@pb.edu.pl; 2 The Main School of Fire Service, Faculty of Security Engineering and Civil Protection, Slowackiego Street 52/54, 01-629 Warsaw, Poland

**Keywords:** bio-based, flame retardant, environmentally friendly, unsaturated polyester resin, epoxy resin

## Abstract

Thermosetting resins are used in many applications due to their great mechanical properties, chemical resistance, and dimensional stability. However, the flammability of thermosets needs to be improved to minimize fire risk and meet fire safety regulations. Some commercially available flame retardants have an adverse effect on people’s health and the environment. Thus, the development of novel, more sustainable flame retardants obtained or derived from biomass has become an objective of contemporary research. The objective of this study is to summarize recent progress on bio-based flame retardants for thermosetting resins so as to promote their prompt development. Groups of biomass compounds with a potential for flame retardant industrial applications were introduced, and their thermal degradation was investigated. The authors focused mostly on the thermal degradation of composites containing bio-based flame retardants determined by thermogravimetric analysis, their tendency to sustain a flame determined by a limiting oxygen index, and fire behavior determined by a cone calorimeter test. The results showed that the mode of action is mostly based on the forming of the char layer. However, in many cases, there is still a necessity to input a high amount of additive to achieve significant flame retardancy effects, which may adversely impact mechanical properties.

## 1. Introduction

The global production of plastics has been growing continuously since 1950 and reached 200 million tons in 2010. In 2017, it increased to 349 million tons, and Europe alone produced 64.4 million tons of plastics. Just one year later, global plastic production increased to 359 million tons, while in Europe, it decreased to a level of 61.8 million tons. Global plastics production achieved its highest ever level of 368 million tons in 2019, whereas Europe’s plastic production declined for another consecutive year to 57.9 million tons.

The newest data report one million tons lower worldwide plastic production in 2020: however, they do not contain recyclate production. The downward trend of plastic production in Europe has been maintained, and the value decreased to 55 million tons. Based on Plastic Europe’s 2021 report, it can be noted that Asia accounted for the largest share, about 49% of global plastics production, with China at 32%, Japan at 3%, and the rest of Asia at a total of 17%. The North American Free Trade Agreement (NAFTA) countries (USA, Canada, Mexico) produced 19%, Europe 15%, Latin America 4%, Middle Eastern Africa 7%, and CIS countries (former republics of the USSR) 3% of the global plastics production volume. Further increases in the production of plastics are predicted, which is related to the increasing scope of their application [1].

The growth in the usage of plastics is because of their numerous beneficial advantages. However, the specific structure of polymers is also a factor limiting their usage, especially when they are exposed to heat. Thermal testing is essential in order to evaluate behavior and qualify for long-term application. The general term “fire hazard” refers to several characteristics, such as ignitability, heat release, ease of extinction, flame spread, smoke release, and toxicity. The rapid spread of fire in vehicles, rolling stock, and buildings may be contributed to by synthetic polymers [2].

Plastics may pose a significant fire hazard in the ground and in solid form. The presence of dust obtained from processing such as cutting, milling, or grinding may lead to a fire or even an explosion [3]. The smoke produced in a plastics fire is also hazardous to people as it impedes evacuation by reducing visibility and exposes them to the aspiration of toxic gases [4]. Due to severe fire hazards and high fuel values of plastics that are commonly used in various areas of human life (such as in the packaging and automotive industries, as well as building and construction), there is a necessity to use flame retardants to meet proper fire standards [5].

**Figure 1:**
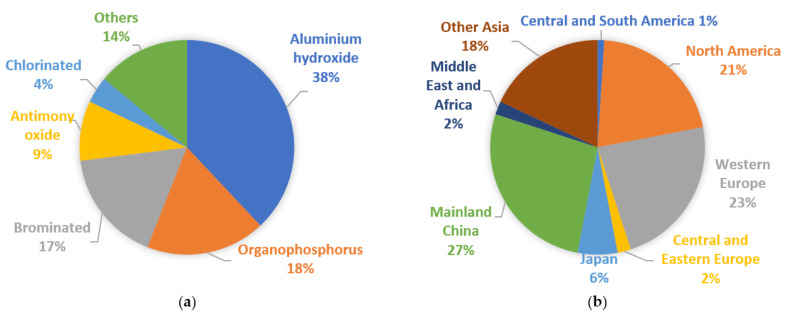
World consumption of flame retardants [7]: (**a**) by type; (**b**) by region.

Over 2.26 million tons of flame retardants (FRs) are produced worldwide annually. With a growth of approximately 3% per year, the estimated market value in 2025 is USD 11.96 billion. According to a 2020 market study by IHS Consulting, the most frequently used FR is aluminum hydroxide. Halogenated FRs account for 21% even though they are forbidden in many countries due to environmental concerns and their adverse effect on human health. However, they are still used in significant amounts, especially in Asia. Research shows that in the Asian region, world consumption of FRs has reached 51%, and in consequence, a considerable amount of brominated and chlorinated FRs are still in use [6]. The world consumption of different types of FRs in 2019 is presented in Figure 1a, and the world consumption of FRs divided into regions is presented in Figure 1b.

FRs are a diverse group of compounds that affect the combustion process of plastics. The addition of FRs may prevent materials from burning, or slow down their combustion, thus protecting the final product. Therefore, FRs play a crucial role in plastics formulation [2].

The incorporation of FRs in polymers reduces heat release rate, flammability, smoke production rate, and overall combustion efficiency. Some FRs are highly effective, even with low content. However, not all groups of FRs are environmentally safe and non-toxic. The replacement of halogenated FRs, organophosphorus FRs, and others, due to the imposed recent restrictions, leads to the development of more sustainable FRs [8,9].

The need for the synthesis of novel bio-based FRs is motivated by green chemistry and its objective of using more renewable and sustainable materials not generated from finite petroleum reserves. The term “biobased” refers to compounds obtained or derived from biomass [10].

Despite the real challenge of preparing FRs from renewable resources, the demand for FRs generated from biomass continues to grow. A fair number of biobased compounds and their derivatives have already been investigated as additives to plastics. However, most of them are not commercially available yet.

The aim of this study is to present and discuss advances in the development of biobased additive FRs for thermosetting resins. Biomass was investigated as a renewable resource to produce biobased flame retardants. Furthermore, the toxicity of its components was described. Recent advances in biobased additive FRs for thermosetting resins are reviewed, and their flammability is described in detail.

## 2. Biomass

Biomass is a huge resource of bio-based materials. Due to its abundant resources and acceptable price, a broad range of chemicals is produced from biomass derivatives. A potential in flame retardancy comes from its high char forming ability [2].

Bio-based resources contain an organic non-fossil carbon derived in less than 100 years from plants, animals, algae, microorganisms, or organic waste streams [11]. Biomass can thus be generally defined as organic material of plant (fitomass), animal (zoomass), or microorganism origin [12,13]. The utilization of fitomass, including wood, forest, yard, and farm waste, was a principal matter of concern until not so long ago. However, in recent years a growing interest in the remaining types of biomass was also observed [13].

**Figure 2:**
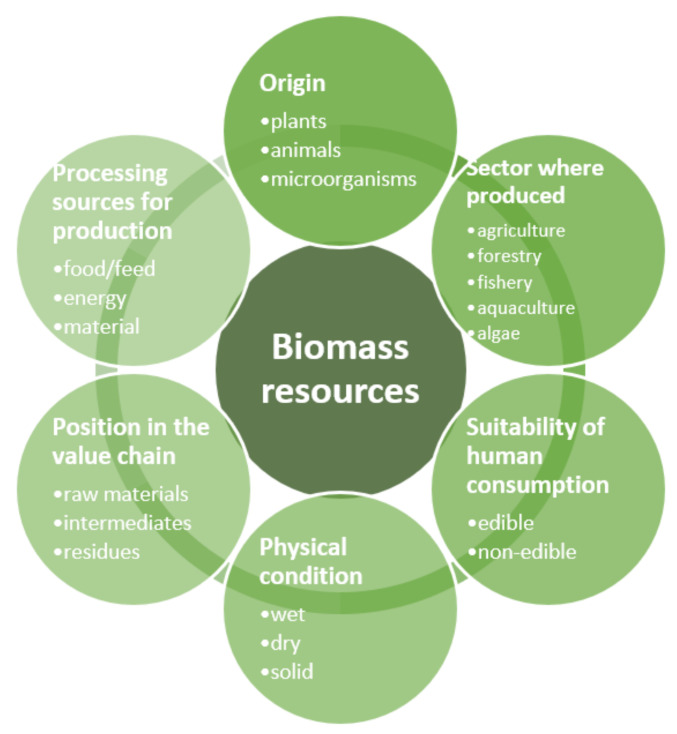
The classification of biomass resources [11].

The aforementioned origin does not form the only criterion for the classification of biomass. Other criteria, such as the production sector, suitability of human consumption, physical condition, position in the value chain, and processing source, are shown in Figure 2.

One of the most frequently used classifications is based on the major components of biomass and refers to edible (starch, sugar, and oil-rich) and non-edible (lignocellulosic) biomass. The major biomass components are shown in Table 1.

Many factors such as geographical location, climate, soil type, or a part of the plant influence the composition of biomass and set conditions for the most suitable thermal treatment. Proximate, elemental chemical, and chemical analyses may be carried out to determine its composition. The former analysis provides information about the volatile matter, fixed carbon, and the percentage of ash. The second analysis informs us about the content of individual elements such as carbon, hydrogen, oxygen, or nitrogen. The latter analysis provides information about the content of different chemical compounds such as cellulose, hemicellulose, lignin, and extractives [14]. Examples of the typical chemical composition of dry plant biomass and dry waste are presented in Table 2. Apart from cellulose, hemicelluloses, and lignin, biomass can also contain ash, proteins, and extractives [15].

## 3. Bio-Based Compounds in the Flame Retardant Industry

**Figure 3:**
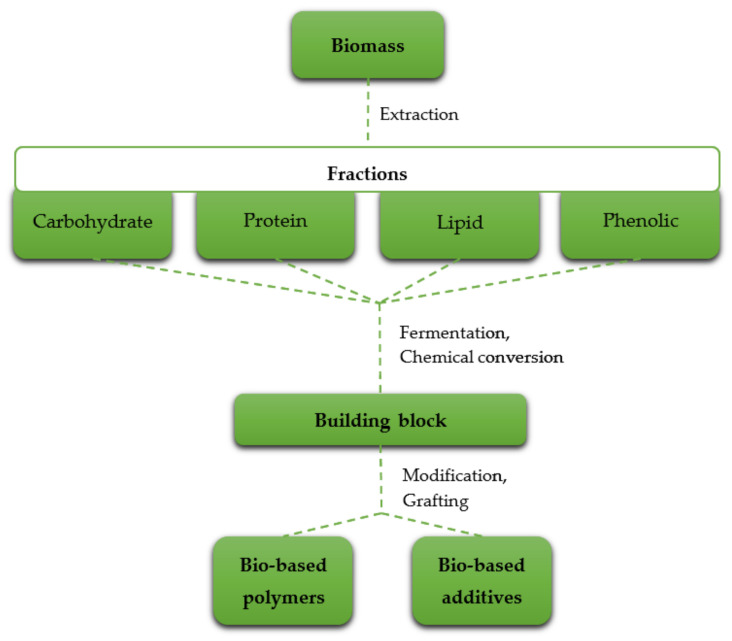
The conversion of biomass to bio-based polymers and additives [10].

The conversion of biomass is essential. The extraction, separation, and modification of biochemical compounds contained in the biomass in various processes are characterized in terms of biorefinery. Four main fractions can be obtained: carbohydrate, protein, lipid, and phenolic. They may be used as such or further modified [10]. The scheme of processes for obtaining different compounds from biomass is presented in Figure 3.

Groups of biomass-derived compounds with the potential to improve flame retardancy of polymers are described in the sections below.

### 3.1. Saccharide-Based Products

**Figure 4:**
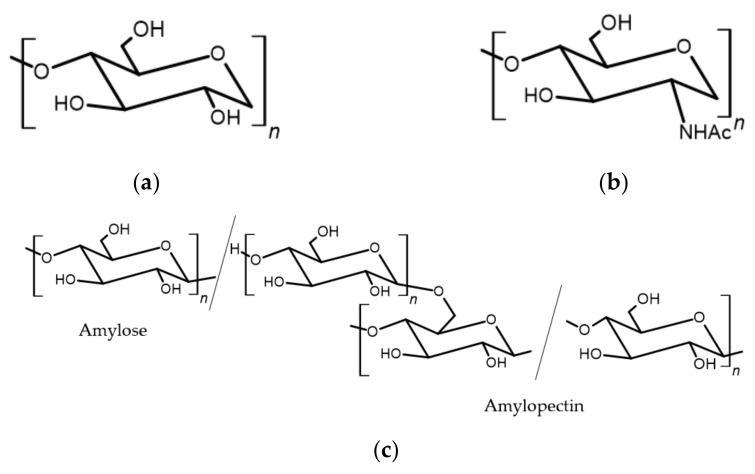
Structures of different polysaccharides [16]: (**a**) cellulose; (**b**) chitosan; (**c**) starch.

Out of all the carbohydrates, polysaccharides have the potential for the development of FRs. Amongst others, the most promising are cellulose, starch, and chitosan [10]. Structures of cellulose, starch, and chitosan are presented in Figure 4.

#### 3.1.1. Cellulose

Cellulose, as the most widespread polymer, is present in all plants and algae. This linear, stereo-regular polysaccharide is built from D-glucopyranosic units connected with 1.4-β-glycosidic bonds. The origin of cellulose influences its degree of polymerization, which varies from 1000 to 30,000 [15].

The thermal degradation of cellulose takes place between 300 °C and 400 °C, with the highest mass loss at ca. 370 °C. This is explained by the breaking of the glycosidic bonds and forming of anhydro-saccharides. A small mass loss is observed below those temperatures according to dehydration reactions [10]. They result in the formation of intermediary compounds called active cellulose or anhydrocellulose. At a temperature of 800 °C, the left char accounts for 15 wt% of the initial mass [10,17].

The degradation of cellulose heavily depends on temperature and pyrolysis time. Lower temperature and lower heating rates favor dehydration reactions resulting in enhanced char production. On the other hand, higher temperatures and faster pyrolisis favor depolymerization, increasing volatile production and decreasing solid residue. Several other factors influence the degradation pathway of cellulose. An increase in the surface area of cellulose, the presence of inorganic salts, and the presence of other biomass constituents, such as small amounts of lignin, intensify charring, whereas the higher crystallinity of cellulose reduces char formation [17].

#### 3.1.2. Starch and Its Derivatives

Starch, present in plants, is a polymer similar to cellulose. It contains two types of macromolecules: amylose and amylopectin. The first one is built of glucose linked through an α-1.4 bond, whereas the latter is a branched polymer linked through an α-1.6 bond [18].

The thermal degradation of starch takes place in three steps. The first one occurs up to 110 °C. Due to the hygroscopic nature of starch, initial weight loss is related to evaporation and physical dehydration. The second step, when the chemical dehydration and thermal decomposition occur, starts above 300 °C. Ether bonds are formed, and condensation inside the glucose ring takes place, resulting in its breakdown. The last step is the carbonization and formation of large, conjugated structures. It takes place at temperatures above 500 °C. The char left accounts for 15–20% of the initial mass [10,17].

The degradation of starch depends mostly on its composition and structure. Higher amylose content decreases the thermal stability. On the other hand, reducing the number of hydroxyl groups by acetylation increases the thermal stability of starch [17].

Due to the large amounts of starch produced, it is often converted into its derivatives, such as dextrins. Cyclodextrin is obtained from starch in the process of bacterial degradation [10]. These cyclic oligosaccharides are made of glucose units linked by α-1,4-glycosidic bonds. Its three forms α, β, and γ, differ by the number of glycosidic bonds, 6, 7, and 8, respectively [19]. The thermal degradation of cyclodextrins occurs in three steps. Below 100 °C, absorbed water and water of crystallization are released. The second step occurs between 250 and 400 °C and is associated with forming the char layer. Above 400 °C, a slow thermal degradation is observed [20].

**Figure 5:**
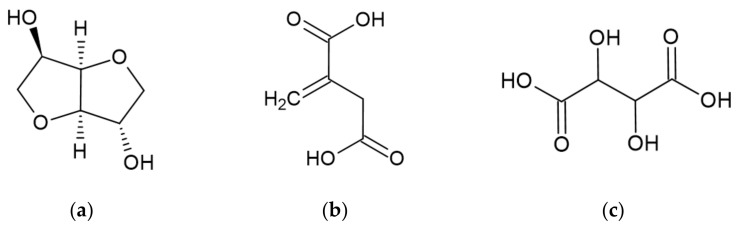
Structures of: (**a**) isosorbide [21]; (**b**) itaconic acid [22]; (**c**) tartaric acid [23].

Other derivatives of starch that could be used in the production of FRs are isosorbide, itaconic, and tartaric acid [10]. Their structures are presented in Figure 5.

Isosorbide is a building block obtained from starch through its hydrolysis to glucose, hydrogenation to sorbitol, and then dehydration to the final product [21]. The thermal degradation of isosorbide takes place in one step in an inert atmosphere as well as in an oxidizing atmosphere. It rapidly decomposes, and the degradation peak reaches its maximum at 200 °C in nitrogen due to evaporation. However, in an air atmosphere, the maximum degradation peak is achieved insignificantly faster at 198 °C. Isosorbide leaves no char residue at 800 °C in an air atmosphere [24].

Itaconic acid may be obtained from starch, glucose, and molasses. However, it is produced commercially through microbial fermentation [25]. Itaconic acid undergoes one-step degradation under a nitrogen atmosphere in a temperature range of 25–400 °C. The main degradation occurs at 169 °C and reaches its maximum at 210 °C. The char residue represents 0.61% of the initial mass [26].

Tartaric acid naturally occurs in grapes, and it is commercially produced mostly from waste products from the wine industry [27]. Thermal degradation of L and D tartaric acid was studied under a nitrogen atmosphere in a temperature range of 300–760 K. The one main degradation step occurred at a similar temperature of 443 K and 443.2 K, respectively. This was established for the release of water and carbon monoxide due to the formula of tartaric acid, which is C_4_H_6_O_6_. The char residue accounts for 5.6% of the initial mass for L tartaric and 5.1% for D tartaric acid [28].

#### 3.1.3. Chitosan

Chitosan is found in a significant amount in fungi and yeasts, crustaceans, and arthropods [17]. It is a copolymer of D-glucosamine and N-acetyl-D-glucosamine linked by a β-1,4 bond [18].

The thermal degradation of chitosan takes place in three steps. In the first step, between 130 and 140 °C, dehydration and the release of loosely bonded water occur. In the next step, between 250 and 360 °C, further dehydration, deacetylation, and depolymerization of chitosan take place. The residual decomposition takes place at above 400 °C, but with a low mass-loss rate. Above 500 °C, the char residue can account for 40% of the initial mass [10,18].

### 3.2. Bio-Based Aromatic Compounds

#### 3.2.1. Lignin

**Figure 6:**
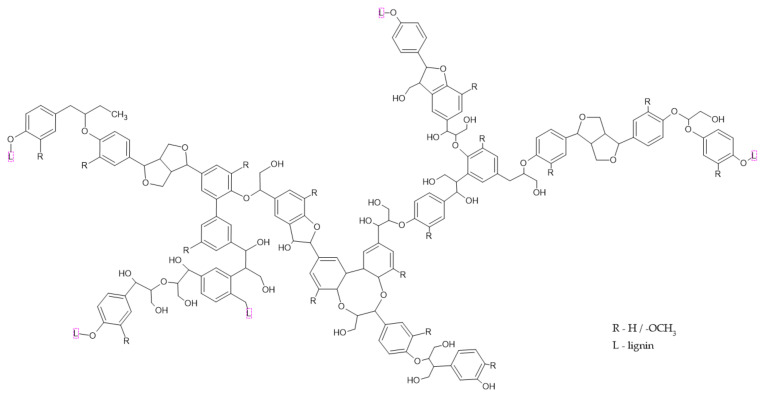
The typical structural model of lignin [30].

Lignin, as one of the most abundant polymers in the world, is an amorphous polymer containing aliphatic and aromatic units. It contains various functional groups such as phenolic, hydroxyl, and carbonyl [29]. It is found in higher plants and some algae [10]. The typical structural model of lignin is presented in Figure 6.

The thermal degradation of lignin differs from other biomass constituents. It occurs in a broad range of temperatures between 200 and 500 °C. The release of water physically bonded and other volatile compounds occur up to 180 °C. The decomposition starts at ca. 200 °C. According to [31], the first step takes place between 200 and 260 °C when the low molecular weight products are released, and the second step occurs between 275 and 400 °C with the breakdown of the main chain. Further condensation of the aromatic structures occurs above 500 °C. However, other studies [32] show that the main decomposition may take place up to 565 °C with a slight division for primary (185–400 °C) and secondary pyrolysis reactions (400–565 °C). The char residue at 600 °C may account for 57% of the initial mass [10].

#### 3.2.2. Tannins

**Figure 7:**
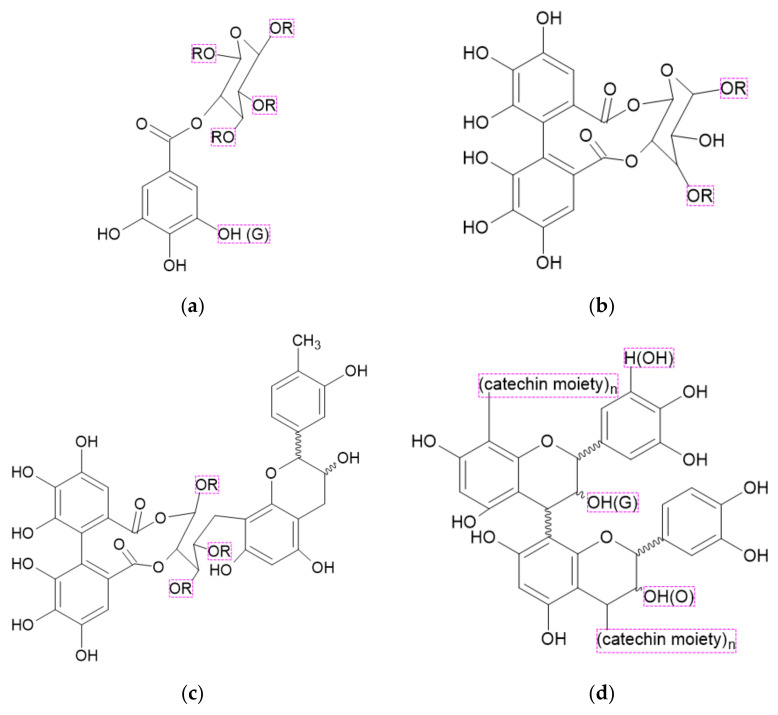
Structures of different tannins [34]: (**a**) gallotannin; (**b**) ellagitannin; (**c**) complex tannin; (**d**) condensed tannins.

Tannins are a class of complex biomolecules synthesized by various plants. Their molecular weights range between 500 and 20,000. They may be divided into hydrolyzable tannins, such as gallo and ellagitannins, condensed tannins when catechin is linked with C4 glycosidic bond to gallo or ellagitannin units, or complex tannins in which a catechin unit is bound glycosidically to gallo or ellagitannin units [33]. Examples of different types of tannin structures are presented in Figure 7.

Generally, the thermal degradation of tannins occurs in three steps. In the first step, between 50 and 170 °C, absorbed water is released. The second step that ends at 400 °C is attributed to the thermal decomposition of organic components. Above this temperature, small molecules are released, and char forming reactions take place. Char residue is higher for condensed tannins than for hydrolyzed ones. However, the thermal decomposition may occur at different temperature ranges depending on the type of tannin [35].

Other aromatic compounds such as phloroglucinol, levulinic acid, or cardanol can also be used in the production of FRs [17].

**Figure 8:**
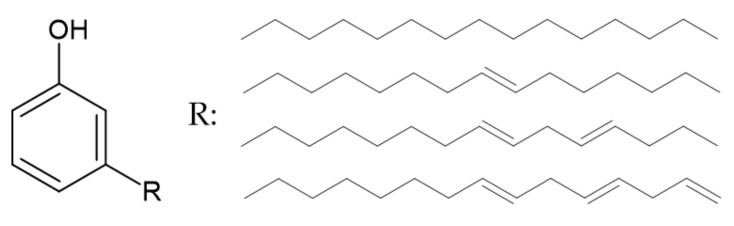
The structure of cardanol [36].

One of the highest yield derivatives of the shell of cashew nut is cardanol [36]. Its structure is presented in Figure 8.

According to [36], thermal degradation of hydrogenated cardanol occurs in one step in an air atmosphere. A weight loss of 96% is observed between 200 and 320 °C, with the maximum at 250 °C. However, according to [37], in the same conditions, thermal degradation may occur in three steps, with several maxima at 270 °C, 427 °C, and 473 °C. The char residue at 800 °C accounts for 2% of the initial mass.

#### 3.2.3. Gallic Acid

**Figure 9:**
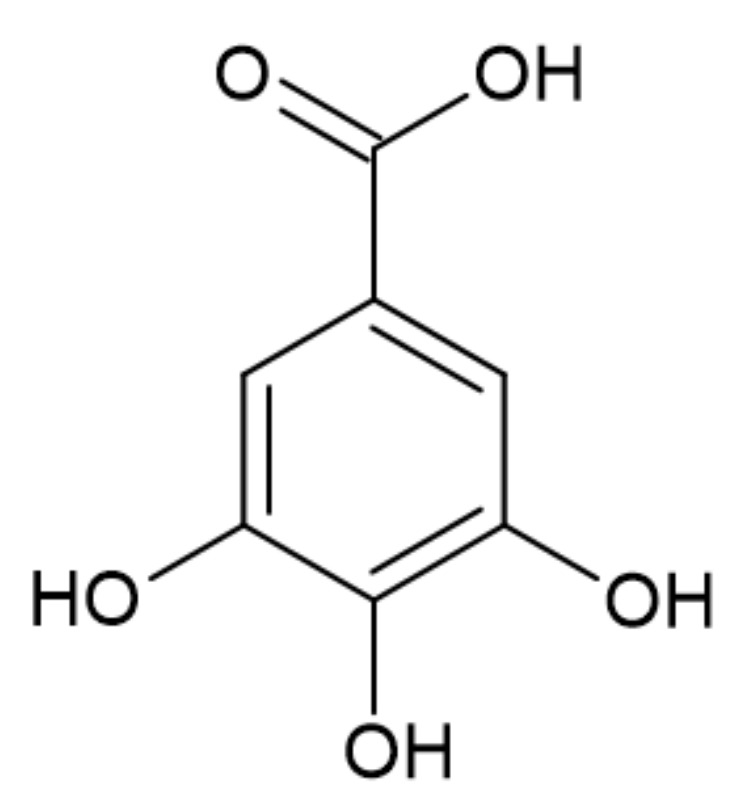
The structure of gallic acid [39].

Gallic acid, one of the simplest biophenols found in many plants, is obtained from the hydrolysis of gallotannins [38,39]. The structure of gallic acid is presented in Figure 9.

The thermal degradation of gallic acid starts at 210 °C. Before, between 74 and 107 °C, the first mass loss is observed according to the release of hydration water. The main degradation occurs in two steps [40]. The mass loss associated with the decarboxylation is observed at 269 °C and then at 327 °C. The char residue at 600 °C and 900 °C accounts for 25.7% and 19.3% of the initial mass, respectively [41].

#### 3.2.4. Ellagic Acid

**Figure 10:**
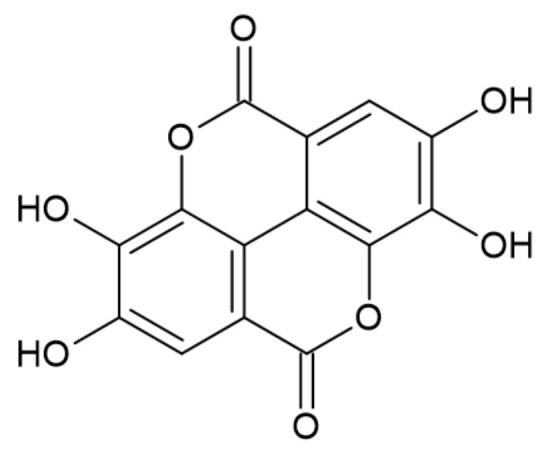
The structure of ellagic acid [42].

Ellagic acid is obtained from the hydrolysis of hexahydroxydiphenic acids contained in ellagitannins. It is found in different berries such as strawberries, blackberries, and raspberries [38]. The structure of ellagic acid is presented in Figure 10.

The thermal degradation of ellagic acid occurs in three steps. The first weight loss occurs at about 100 °C and corresponds to the release of the attached water. The main degradation starts at 400 °C reaching its maximum at 496 °C. The second and the third degradation steps are associated with the pyrolysis of lactone groups, and they achieve their maxima at 528 °C and 630 °C, respectively. The char residues were greater compared to gallic acid. They accounted for 52.9% and 32.7% of the initial mass at 600 °C and 900 °C, respectively, which is due to the different structures of these two acids. Ellagic acid contains two aromatic rings in its structure, and thus its charring ability is better [41].

Although ellagic acid could be a great material for the production of bio-based FRs, its high price and poor market availability hamper its practical application [43].

### 3.3. DNA

**Figure 11:**
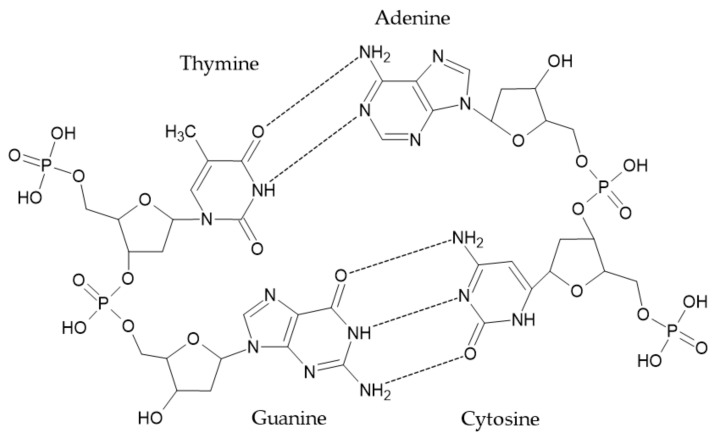
The molecular structure of DNA [17].

DNA is an important polymer carrying genetic information. It consists of two double-ring purines (adenine and guanine) and two single-ring pyrimidines (cytosine and thymine) attached to deoxyribose sugar and phosphate group [44]. The structure of DNA is presented in Figure 11.

Thermal degradation of DNA takes place in several steps. The first step occurs up to 100–150 °C when absorbed water is released. In a temperature range of 150–200 °C significant physical change is observed, although the release of combustible volatiles is low. The cell-based structure obtained between 200 and 400 °C is disrupted by the formation of an open cell-based structure. At this stage, the largest amount of combustible volatile compounds are released. Above 400 °C progressive chemical restructuration takes place. The char residue at 700 °C can account for 27–48% of the initial mass [45]

However, despite the development of a large-scale method for DNA production, its cost is still significantly higher than that of commercial FRs [46].

### 3.4. Proteins

Proteins are polymers consisting of hundreds to thousands of amino acids linked with a peptide bond. They are found in all living organisms [47]. Caseins and hydrophobins exhibit great potential as FRs [17].

**Figure 12:**
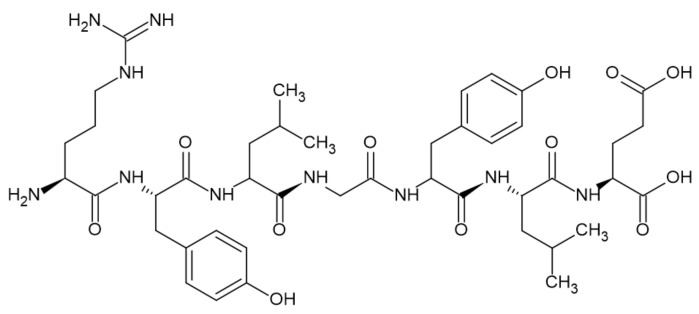
The structure of casein [48].

Casein is a milk protein built of four protein subunits, α-s1, α-2, β, and κ, whose size is between 11,500 and 25,000 Daltons [48]. The structure of casein is presented in Figure 12.

The thermal degradation of casein occurs in five steps in an air atmosphere and four steps in a nitrogen atmosphere. In the first step, which is similar in both, between 46 and 172 °C in air and between 45 and 176 °C in nitrogen, the release of water is observed. The proper degradation starts above these temperatures. In the air, steps of degradation occur between 172–238 °C (endothermal) and 238–426 °C, and 426–642 °C and 642–771 °C (exothermal). In this case, no residue is observed. In nitrogen, steps of degradation occur between 176–248 °C, 248–380 °C, and 380–610 °C, with all being endothermal. The residue accounts for 20% of the initial mass [49,50].

Hydrophobins consist of cysteine-rich proteins with a low molecular mass of 7000 to 15,000 Daltons. Eight cysteine residues form four disulfide bonds to stabilize its structure, which is specific for hydrophobins. They are mainly produced by filamentous fungi [51,52]. Their disulfide bonds degrade at ca. 200 °C, which results in the release of gaseous H_2_S, therefore giving rise to char formation [51].

### 3.5. Phytic Acid

**Figure 13:**
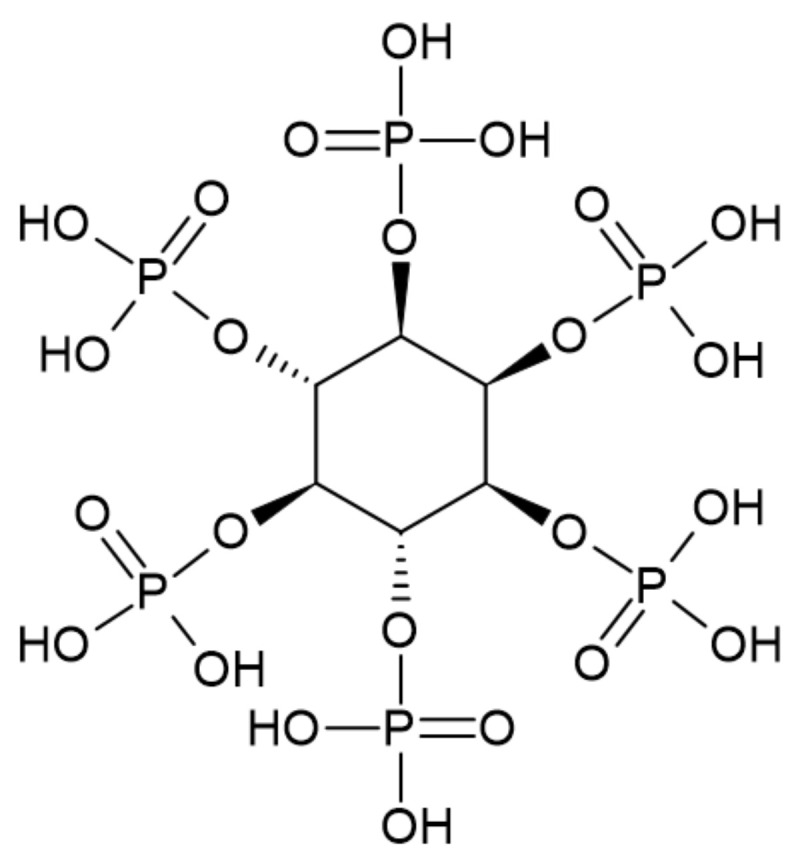
The structure of phytic acid [53].

Phytic acid, known as the inositol hexakisphosphate, comprises 6 phosphate and 12 hydroxyl groups. It may bond with various metal ions and positively charged compounds. The molecular structure of phytic acid is presented in Figure 13. Phytic acid can be extracted from beans, legumes, oilseeds, and cereal grains [53].

Thermal degradation of an aqueous solution of phytic acid occurs in four stages. The first step takes place between 40 and 160 °C when water present in the solution is released, and the carbonization starts. The next steps occur between 162 and 292 °C and 248 and 447 °C. They may be attributed to the carbonization and dehydration reactions. The last step occurring between 447 and 863 °C corresponds to the thermal decomposition of phytate groups and elimination of elemental carbon. The residue accounts for 3% of the initial mass [54].

## 4. The Toxicity of Bio-Based Compounds

Some of the currently used FRs have an adverse or unknown effect on human health and the environment. The use of biomacromolecules in flame retardancy can be highly desirable due to their overall low environmental impact and toxicity [55]. Tannins reduce rumen protein degradation and thus diminish. However, not all biomaterials are safe.

Cellulose as a bulk material is proven to be safe for humans and the environment. However, its properties cannot be directly related to cellulose nanomaterials. In spite of the fact that nanomaterials do not exhibit immediate adverse effects on human health and the environment, according to a recent review by Environmental, Health, and Safety (EHS) on issues of bio-based nanomaterials, there are several cases of materials that are not inert and may interact with their surroundings. Cellulose nanofibrils and nanocrystals are non-toxic in general. Only slight indications of their toxicity and, in some cases, inflammatory response were reported [56].

Starch is a non-toxic, biodegradable, and relatively inexpensive biopolymer [57]. Nowadays, there is growing interest in starch-based nanoparticles due to their various potential applications, such as additives, stabilizers, sorbents, or catalysts. Although they exhibit less damage to normal cells, starch-based nanoparticles may easily deposit in human organs [58].

Chitosan is a relatively non-toxic and biocompatible polymer, and it is often used in industry. Due to its antimicrobial features, it is also used in the health industry [59]. However, its modifications may be less or more toxic. Purity is a key aspect because proteins, metals, and other contaminants may have an adverse effect on further syntheses. Any residual reactants have to be removed before application in order to avoid their cytotoxicity [60]. The toxicity of chitosan is dependent on the degree of deacetylation and its molecular weight [61]. In addition, chitosan nanoparticles may be even more toxic than chitosan itself [62].

Lignin materials are generally described as biodegradable and environmentally friendly. They may act as antimicrobials, antioxidants, stabilizers, and natural adsorbents for heavy metals [63]. However, it is reported that lignin, being a major by-product of the pulp and paper industry, may have a toxic effect on benthic and plankton zones, decreasing the diversity of zoobenthos and phyto–zooplankton [64].

Depending on the chemical structure, source and levels, tannins may have a positive or an adverse effect on human or animal health and the environment. Tannins may form indigestible complexes with proteins, which may result in a reduction in the feed intake by ruminants. On the other hand, condensed tannins may increase the nutritional value of feed and its quality when present at low-to-moderate concentrations. Tannins reduce rumen protein degradation and thus reduce NH_3_ and N_2_O release, which are important in the context of environmental pollution, and increase the nitrogen retained in soil [65]. Complexes with proteins are also postulated to cause toxicity to bacteria. When comparing hydrolyzable and condensed tannins, the latter is found to be more toxic due to their degradation to gallic acid [66].

Proteins and nucleic acids are environmentally sustainable and may provide an alternative to traditional FRs [46].

Phytic acid is a biocompatibile material. It is non-toxic and widely applied as an antioxidant and in anticancer formulations [67].

Even though most of the biomacromolecules are safe for humans and the environment, it is important to consider all derivatives individually. As can be seen, the chemical structure, molecular weight, ability to form complexes, as well as particle size may influence the toxicity of bio-based compounds.

## 5. Bio-Based Flame Retardants for Thermosetting Resins

Thermosetting resins are polymers forming a highly cross-linked network after curing. They find application in many fields such as coatings, adhesives, the electronics industry, or composites due to their great dimensional stability, chemical resistance, and mechanical properties [68].

However, due to the high flammability of thermosetting resins, it is crucial to improve their flame retardant properties. Generally, there are two types of FRs, additive and reactive. The former is mixed with polymers by physical blending, while the latter is chemically bonded with a polymer network [69,70].

### 5.1. Bio-Based FRs for Epoxy Resin

**Figure 14:**
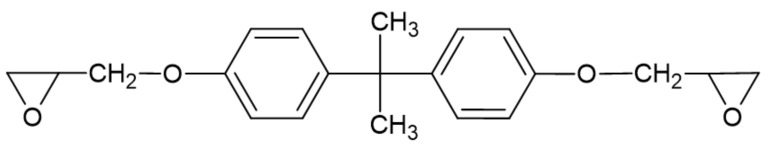
Idealized structure of epoxy resin based on DGEBA [72].

One of the most commonly used resins worldwide is epoxy resin (EP). Its structure is based on bisphenol A diglycidil ether (DGEBA) [71]. EP consists of long-chain molecules with reactive sites at both ends and ring groups in their center. The presence of aromatic structures contributes to the great mechanical properties of EP due to better absorption of mechanical and thermal stresses than that of linear groups [72]. An idealized structure of epoxy DGEBA resin is presented in Figure 14.

Since the first commercial interest arose in the 1940s, EPs have proved to be superior among all thermosetting resins because of their high tensile strength/modulus, low shrinkage on cure, dimensional stability, and chemical inertness even though they have never been the cheapest [73,74]. Currentky, EP is frequently used as a polymeric matrix in the development of high-performance materials in applications where thorough, durable coating and adhesives are needed in the electrical industry and commercial and military aircraft industries [71].

However, the main disadvantage of EPs is that they are easily flammable, making them impossible to use in applications when fire-resistant materials are required. Additive or reactive FRs must be used to reduce their flammability.

**Figure 15:**
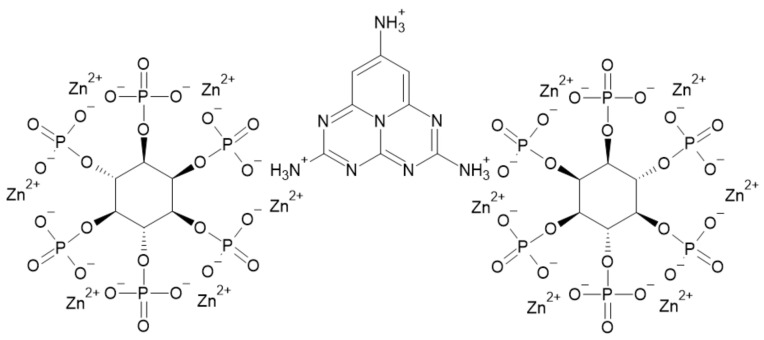
The structure of the (g-C_3_N_4_/PHZn) [75].

Zhang et al. [75] studied the effect of core-shell graphitic carbon nitride/zinc phytate on EP. The (g-C_3_N_4_/PHZn) was synthesized by calcination and chemical precipitation from melamine, phytic acid, and zinc nitrate hexahydrate. The structure of the FR is presented in Figure 15.

The thermal degradation of (g-C_3_N_4_/PHZn) occurred in two stages. Between 250 and 350 °C, phosphonate groups underwent dehydration and condensed into polyphosphoric compounds. Afterward, from 550 to 650 °C, further degradation into oxides took place, and the graphitic carbon nitride degraded. The char residue accounted for 43.42% of the initial mass.

The thermal degradation of the EP/(g-C_3_N_4_/PHZn) composite was similar to pure EP. However, pure EPs exhibited one degradation step, whereas the composite had a second degradation stage at ca. 600 °C, which corresponded to the degradation of the additive. The temperature at the mass loss of 5 wt% (T_5_) fell from 365.3 to 346.7 °C. The maximum mass loss rate fell by 3.88%/min, and the char residue was higher by 5.22 wt%.

The Limiting Oxygen Index (LOI) of a composite containing 5 phr (parts per hundred rubber) of (g-C_3_N_4_/PHZn) increased from 24.5% for neat composite to 28.3%, thus reducing the fire hazard. The peak heat release rate (PHRR) decreased by 71.38%, and the total heat release (THR) decreased by 58% compared to neat resin. The time to ignition (TTI) was prolonged, and the fire growth index (FGI) was lower. The barrier and labyrinth effect of g-C_3_N_4_ nanosheets improved flame retardancy as well as total smoke production (TSP), which was 0.10 m^2^ lower for the (g-C_3_N_4_/PHZn) compared to pure resin. However, due to poor adhesion strength, the tensile strength, elongation at break, and impact strength were reduced by 61%, 44%, and 51%, respectively [75].

The effects of the addition of chicken eggshell to an intumescent flame retardant system based on ammonium polyphosphate (APP), pentaerythritol (PER), and melamine (MEL) were studied by Xu et al. [76]. The thermal degradation of chicken eggshells occurred in three steps. In the first step, between 350 and 450 °C, the thermal degradation of organic compounds took place. Due to strong interaction with calcium carbonate, which accounts for 97% of the initial mass, some organic compounds decomposed up to 600 °C. Between 600 and 830 °C, this main component underwent thermal degradation with the production of calcium oxide and calcium dioxide. The char residue accounted for 50% of the initial mass at 900 °C. The T_5_ of pure EP was 148.3 °C, and the addition of an intumescent system with 3% of eggshell increased its value to 209.7 °C, improving its thermal stability. However, the temperature at the maximum mass loss rate was significantly reduced from 441.3 °C to 362.3 °C. The char residue at 800 °C was significantly higher for the composite and reached a value of 22.4 wt%.

The LOI value of coating containing 40% of the intumescent system increased from 19.2% for pure epoxy to 29.1%. The incorporation of 3 wt% of eggshell further improved the LOI value up to 31.5%. The PHRR was reduced from 1293.3 kW/m^2^ to 181.3 kW/m^2,^ and THR was reduced from 86.9 MJ/m^2^ to 33 MJ/m^2^ for that composite, showing improvement in flame retardancy in comparison to neat resin. The addition of eggshell also enhanced smoke production. The total smoke release (TSR) was reduced from 1214.7 m^2^/m^2^ to 645.8 m^2^/m^2^. It was 15% lower compared to coating without the additive. The smoke density rating was 45% lower, resulting in an improvement in fire safety. The synergistic effect of eggshell and the standard intumescent system was due to the reaction of additives with volatile phosphorus. Calcium phosphate and calcium metaphosphate are formed, which block heat and mass transfer between gas and condensed phases, improving flame retardancy and smoke suppression.

Liu et al. [77] investigated the effect of cobalt alginate, synthesized from sodium alginate and phosgene on flame retardancy of EP. The thermal degradation of EP/cobalt alginate (CA) composite did not differ much at a lower temperature zone from neat epoxy. The T_5_ of pure EP was 347 °C, and it was slightly decreased by 10 °C for an EP/CA composite. However, at higher temperatures between 390 and 700 °C, the thermal stability of EP/CA is higher. The char residue at 700 °C accounted for 34.7% of the initial mass.

The LOI value of composite containing 14.56% of CA increased from 20.9% for EP to 24.3%, which showed a slight improvement in the flammability. Although there was no change in time to ignition (TTI), the PHRR was reduced by 670 kW/m^2^ (56.2%) regarding pure EP. However, the THR was slightly higher for EP/CA. The fire growth rate index (FIGRA), significant for fire risk assessment, was considerably lower. It was reduced from 11.8 kW/m^2^s to 3.9 kW/m^2^s. These characteristics indicate that CA reduces the fire risk, but the formed char is not durable and slows the fire instead of preventing it from completion. The TSP is reduced for composites containing 3% of CA by 17.8% compared to EP. However, if the amount of additive is too low, the thin char layer cannot prevent it from releasing gases, and thus TSP is higher. Smoke density tests showed higher light transmittance at 120 s of combustion, which may result in better visibility during a fire. The study on the mechanical properties of EP/CA composites showed that tensile strength, tensile modulus, and flexural strength are lower, but the impact strength is higher than for pure EP.

**Figure 16:**
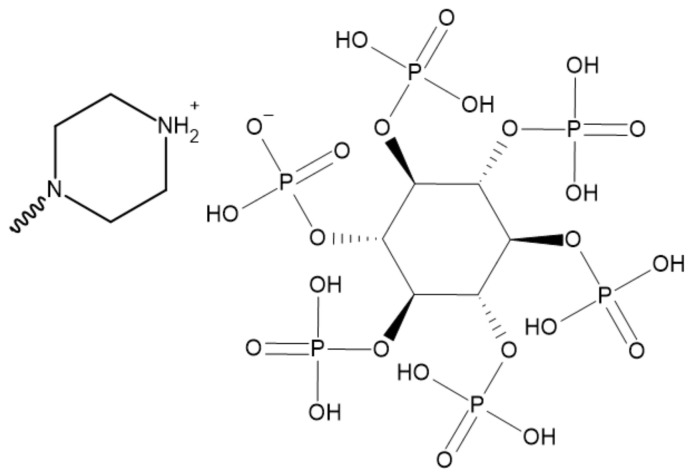
The structure of the piperazine phytate [78].

Huang et al. [78] reported piperazine phytate (PIPT) synthesized from phytic acid and piperazine as a bio-based nitrogen-phosphorus FR. The structure of PIPT is presented in Figure 16.

The thermal degradation of EP/PIPT started faster compared to pure EP. T_5_ was reduced from 384.2 °C to 336.9 °C, and the temperature at the maximum mass loss rate was also reduced and changed from 402.9 °C to 364.3 °C due to the early decomposition of PIPT. However, the char residue was higher and increased from 14.4% to 26.2% of the initial mass because of the catalytic effect of phosphorus-containing acids enabling char formation.

The addition of PIPT increased the LOI value from 23.5% to 35.5% compared to pure EP. The incorporation of additive decreased PHRR and THR by 51.4% and 35.9%, respectively. PIPT showed oneself as an effective smoke suppressant. It reduced peak smoke production rate (PSPR) by 49.7% and total smoke release (TSR) by 44.9% in contrast to pure EP. The high amount of char residue standing for 36.2% of the initial mass suggests good char forming ability. The FIGRA is also lower for EP/PIPT composites resulting in better fire safety of composites than for pure EP. Smoke density, according to a decrease in smoke production, was also lower for EP/PIPT composites. However, mechanical properties such as tensile strength and elongation break are decreased up to 13.8% and 24.7%, respectively.

The incorporation of fly ash (FA) (a by-product from thermal power plants) into EP was investigated by Anh et al. [79]. Fly ash consists mainly of inorganic contents, such as silica, alumina, and calcium oxide. Pure FA, FA modified with NaOH, and FA modified with HCl were investigated. The research showed that with the increase in the additive, the improvement of flame retardancy is achieved according to the burning rate. The lowest burning rate was achieved for EP/FA modified with HCl composite (11.51 mm/min), whereas for EP/FA modified with NaOH composite, it was higher (13.45 mm/min). An EP/pure FA composite achieved a burning rate of 16.3 mm/min. Similar effects were observed for the LOI value. LOI also increased with the increase in the additive content. The highest LOI was achieved for EP/FA modified with HCl composite (23%), lower for EP/FA modified with NaOH composite (22.4%), and the lowest for EP/FA composite (21.4). The improvement in flame retardancy of composites containing modified FA is due to better contact and dispersion in a polymer matrix. The authors conclude that the addition of FA reduces HRR, SPR, and TSP. However, there are no attached results for the cone calorimeter tests conducted (CCT).

Increasing the content of FA insignificantly reduces flexural, tensile, and impact strength. However, it is still higher for EP/FA modified than for EP/FA unmodified. On the other hand, the compressive strength increases with the amount of additive and is higher for EP/FA modified. In this research, the authors did not compare the results obtained with the values for pure EP.

**Figure 17:**
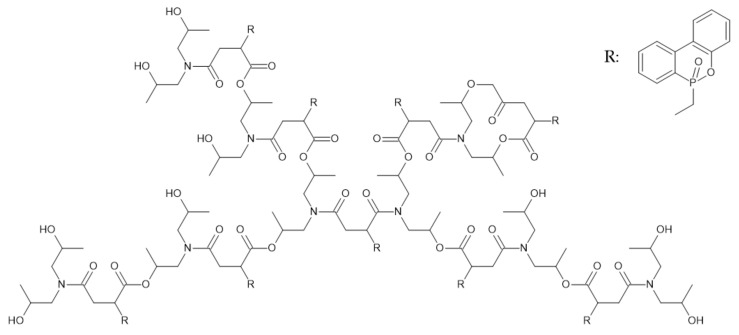
The structure of the ITA-HBP [80].

Zhang et al. [80] studied the effect of the hyperbranched polymer ITA-HBP synthesized by employing itaconic anhydride and DOPO (9,10-dihydro-9-oxa-10-phosphaphenanthrene-10-oxide). The structure of ITA-HBP is presented in Figure 17.

The thermal degradation of ITA-HBP occurs between 220 and 456 °C. T_5_ was lower for all investigated EP/ITA-HBP composites and was reduced from 360 °C to even 311 °C for composites with 20 phr of additive. The char residue accounted for 8.28% of the initial mass at a temperature of 700 °C. A significant amount of produced volatile compounds indicate the gas phase mechanism of flame retardancy. EP/ITA-HBP composites degraded similarly to pure EP in two major steps but at a lower temperature range. The higher amount of char residue (2.88%) of EP/ITA-HBP composite compared to pure EP (1.23%) also indicated a condensed phase mechanism due to forming of the char layer and inhibiting heat and oxygen permeation.

The LOI value of pure EP was 26.4%, whereas the incorporation of 5 phr of ITA-HBP increased this value to 36.4%. Further increase in the amount of additive resulted in an increase in LOI up to 42%. The results of UL-94 tests indicated better self-extinguishing behavior of modified EP compared to neat EP.

The CCT results showed significant improvement in the flame retardancy of EP/ITA-HBP composites. PHRR and THR were reduced from 678.7 kW/m^2^ and 157.9 MJ/m^2^ to 468 kW/m^2^ and 110.2 157.9 MJ/m^2,^ respectively. The TTI was shortened by 18s for the lowest amount of additive (5 phr) and even 30 s shorter for the highest amount of additive (20 phr). This is due to the release of free radicals released during decomposition, promoting the pyrolysis of the epoxy matrix. Volatile compounds released such as CO_2_, N_2_, NH_3_, and NO_2_ diluted the concentration of ignitable gases and cut off the supply of oxygen. The TSR was reduced from 14.441 m^2^/m^2^ up to 11.159 m^2^/m^2^.

Generally, mechanical properties were improved for EP/ITA-HPB composites compared to pure EP. However, impact, flexural strength, and toughness increased and then decreased with the increasing content of additive. The best performance was achieved for the composite containing 10 phr of ITA-HPB, and after further addition of an additive, all the mechanical properties decreased.

The incorporation of lychee peel into EP was researched by Nguyen [81]. This carbon-rich additive slightly increased the LOI value from 20.6% to 21.5% compared to neat resin. However, according to ISO 4589–2:2017, the EP/lychee peel composite achieved the classification of self-extinguishing but was not non-combustible. The results of UL94 tests indicated improvement in flame retardancy of modified EP. The burning rate decreased from nearly 30 mm/min to 23.45 mm/min.

Generally, mechanical properties were improved. Tensile strength increased by 20%, the compressive strength increased by 26%, and impact strength increased by 22%. Only flexural strength was lower by 9% compared to pure EP.

**Figure 18:**
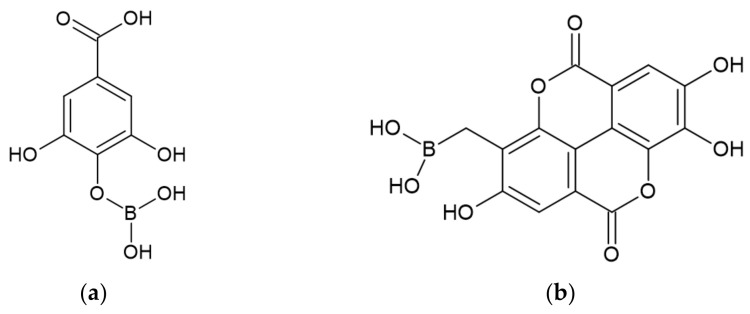
Putative structures of derivatives of acids [41]: (**a**) gallic acid derivative (GAD); (**b**) ellagic acid derivative (EAD).

Karaseva et al. [41] studied the effect of gallic (GA) end ellagic acids (EA) as well as their borate forms on the flame retardancy of EP. Structures of gallic and ellagic acids are presented in Figure 9 and Figure 10, respectively, whereas putative structures of its derivatives with boric acid are presented in Figure 18.

GAD and EAD exhibited improved thermal stability compared to pure GA and EA. Its major decomposition step occurred at 528 °C and 628 °C for GAD and EAD, respectively, due to pyrolysis of carboxylic and lactone groups.

The CCT results show that all additives reduce TTI, PHRR, and THR and increase remaining char residue. Despite the higher thermal stability of GAD and EAD, the TTI was reduced from the 90s for pure EP to 66s for EP/GA composite. However, this composite showed the poorest improvement in the PHRR, which was reduced by 12%. PHRR was 65% lower for the EP/GAD composite compared to neat EP. It is an interesting fact that the addition of GA, EA, and GAD to EP resulted in a change in the HRR curve. The rapid achievement of PHRR and then decrease in the curve indicated that in the early stage, a protective layer was formed. The char residue was higher for all composites and was the highest for EP/GAD composite (13.7% of the initial mass) compared to pure EP (3.9%).

The EP/EA composite exhibited the highest reduction in THR, from 27.0 kJ/g for pure EP to 23.5 kJ/g. Similar results were obtained for EP/GA and EP/GAD (23.9 kJ/g).

### 5.2. Bio-Based FRs for Unsaturated Polyester Resin

**Figure 19:**
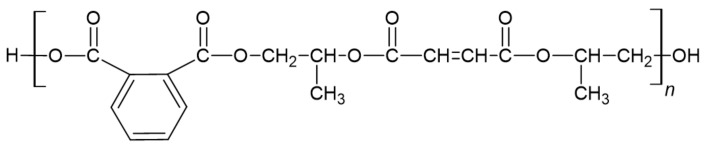
The structure of orthophthalic UPR [83].

Unsaturated polyester resins (UPRs) are viscous, pale yellow solutions of unsaturated polyester oligomeric polyesters of a low degree of polymerization in an unsaturated diluent, mostly styrene. Unsaturated groups in the polyester and those of the reactive diluent undergo addition copolymerization and form a cross-linked network [82]. The schematic structure of orthophthalic UPR is presented in Figure 19.

Since the 1930s, UPRs have constituted a system of major importance in various applications. This is due to the possibility of achieving thermoset articles with desired chemical and mechanical properties. The selection of proper diacids, diols, cross-linking agents, initiators, and other additives, as well as added fillers and reinforcement, affect their end product characteristics [84]. UPRs are commonly used as matrices in composites, of which glass fiber reinforced polyesters are of the greatest significance.

However, UPRs are highly flammable and release significant amounts of smoke and toxic gases during burning. Therefore, to meet the requirements of fire safety regulations, it is crucial to incorporate FRs into resin [85].

A limited amount of literature has been published on bio-based FRs for UPR in contrast to EP.

**Figure 20:**
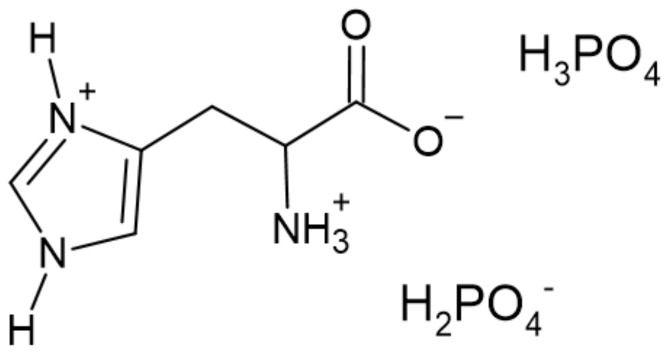
The structure of the L-histidinium dihydrogen phosphate–phosphoric acid [86].

Sałasińska et al. [86] studied the effect of L-histidinium dihydrogen phosphate–phosphoric acid (LHP), synthesized from L-histidinium and phosphoric acid on the flame retardancy of UPR. The structure of LHP is presented in Figure 20.

The thermal degradation of UPR/LHP composites started earlier than for pure UPR and increased with the increase in the additive. This is due to the faster decomposition of organic compounds accompanied by phosphoric acid. The cyclization of shorter chains resulted in the formation of thermally stable aromatic compounds. The main degradation occurred in two steps, distinct from neat UPR that degrades in one major step. These two steps correspond to the decomposition of both FR and polymer. This process was also slower for UPR/LHP composites compared to pure UPR. Pure EP exhibited a T_5_ of 278 °C. However, for UPR/LHP composites, it was lower, and it decreased with the increase in additive. The char residue was higher in all composites researched, reaching 4.3% of the initial mass at 1000 °C, whereas it was only 1.4% for UPR.

A horizontal burning test was used to determine the resistance of materials to flame. The end of a specimen was subjected to a 50 W test flame tilted at 45°. Results showed that the small addition of LHP to UPR (10 wt%) resulted in a reduction in the linear burning rate and a decrease in the intensity of the process. Further increase in the amount of LHP leads to self-extinguishing behavior.

The TTI value was reduced for UPR/LHP composites, which is due to the lower thermal stability of these composites compared to pure UPR. However, a slightly higher value was observed for the sample with the highest loading of additive. It is noteworthy to mention that the TTI depends on several material properties, and thus it may not be as reliable as HRR, for example.

Flattened HRR and reduction in PHRR by even 75% were observed in CCT results for all samples. The THR was also reduced from 159 MJ/m^2^ for pure UPR to 59 MJ/m^2^ according to char formation followed by incomplete combustion. The TSR was significantly reduced by 49%, and the smoke density was reduced by 31% compared to pure UPR. In addition, the highly effective char layer prevented the release of incomplete combustion products resulting in better fire safety.

Chitosan and its derivatives are a point of interest in improving the flame retardancy of UPR. Chen et al. [87] studied the effect of phosphorylated chitosan-coated carbon microspheres (PCCCM) on the flammability of UPR.

The LOI value slightly increased from 19.8% for pure UPR to 21.7% for the composite containing 2 wt% of PCCCM. A further increase in the amount of additive improved LOI value up to 22.2%.

The patterns of the TG curves in nitrogen and air atmosphere after the addition of PCCCM were not significantly different. However, there was a visible change in the residue mass at 800 °C. It increased from 9.3% to 13.3% in nitrogen and from 0.1% to 1.7% in air compared to pure UPR. In addition, results of a TG analysis revealed that PCCCM has a better char forming ability than carbon microspheres.

An earlier igniting effect was observed for UPR/PCCCM composite: TTI decreased by 13s, and PHRR reduced by 18.9%. The THR between 160 and 402 was lower than for neat UPR. However, considering the whole test, the THR was slightly increased from 123.7 kW/m^2^ to 126.9 kW/m^2^.

The PSPR for UPR occurred at 224s and reached 0.34 m^2^/s, and for UPR/PCCCM, it occurred at 316s and reached a lower value of 0.31 m^2^/s. The TSP was significantly reduced by 15.4%, showing improvement in fire safety.

**Figure 21:**
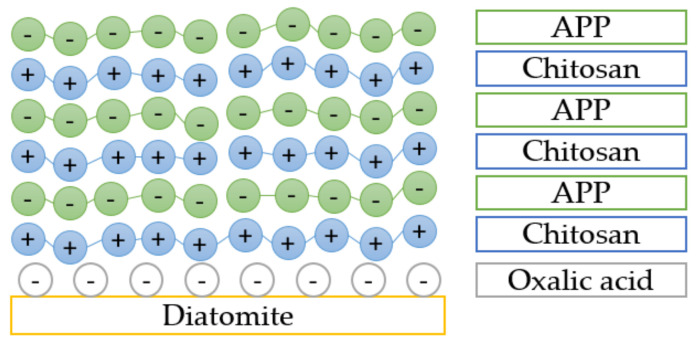
The schematic structure of LbL assembled diatomite based on chitosan and APP [88].

Layer-by-layer assembled diatomite based on chitosan and APP (DCHAPP) was investigated by Chen et al. [88]. The LbL technique is presented as an environmentally friendly FR fabrication method. The pore structure of diatomite was coated by chitosan (cationic layer) and APP (anionic layer). A schematic representation of FR is presented in Figure 21.

The thermal degradation of composites revealed two main degradation steps, with the first one occurring between 250 and 380 °C ascribed to water dehydration and the initial decomposition of APP. Compared to pure EP, a gradual increase in the initial degradation temperature was observed. In the second step, between 380 and 450 °C, the chain scission of UPR and the degradation of APP and chitosan were observed. The maxima of mass loss rate showed better thermal stability of UPR/DCHAPP composites. The increase in char residue was also higher, according to the intumescent coating formed from chitosan acting as a carbonizing agent and APP acting as a blowing agent and an acid source.

The study also contained tests conducted for a composite containing diatomite, APP, and chitosan directly added into a polymer matrix. The results showed an increase in LOI value from 19.3% to 25.7% for an LbL assembled composite with nine bilayers compared to pure UPR. A slightly improved LOI value was also observed for composite with a directly added mixture (23.1%).

The vertical burning test slightly differs from the horizontal burning test. The specimen is placed in a vertical position, and the burner is applied at its free end. The LbL assembled composite achieves a V-0 rating according to the vertical burning test, which accounts for its self-extinguishing and non-drip performance. However, the composite with a directly added mixture achieves the V-2 rating, which accounts for flame extinction within 10s with dripping.

The CCT results showed that UPR/DCHAPP has better flame retardancy than the UPR/mixture. The PHRR and the THR were significantly reduced by 41% and 18%, respectively. Inhibition of combustion was also observed: the TTI was elongated by 5s, which thus affected the SPR. The TSR was significantly reduced from 13,019 m^2^/m^2^ to 6198 m^2^/m^2^.

The addition of DCHAPP seemed not to impoverish the mechanical properties. The flexural and tensile strength was significantly improved due to better smoothness of the surface after deposition of the DCHAPP.

A study on functionalized lignin was conducted by Farishi et al. [89]. Lignin was modified using polyethyleneimine and diphosphorus pentaoxide to form PN-lignin.

The thermal degradation of PN-lignin composite started at 170 °C, whereas lignin started to degrade at 277 °C. Thus, earlier produced char protects the material and prevents heat transport to the inside. However, char residue mass was not significantly higher, which may be due to a smaller number of attached side groups.

A vertical burning test showed that the burning rate [mm^3^/s] was reduced from 64.18 to 43.66 compared to pure UPR, which showed better flame retardancy properties of a composite.

The thermal stability of UPR/PN-lignin composites was slightly improved. Its decomposition starts at 335 °C, which is 9 °C higher than for pure UPR.

Zhang et al. [90] studied the effect of hemp fiber modified with phytic acid (HFP) and mixed with melamine cyanurate (MC) on the flammability of UPR composites.

Thermogravimetric analysis showed better thermal stability of composites containing HFP than HP compared to pure UPR. The initial decomposition temperature was increased by 19 °C. Due to the higher pyrolysis temperature of MC than that of the HF, UPR/HF/MC and UPR/HFP/MC composites revealed worse thermal stability, and the pyrolysis started faster. The char residue at 700 °C was the highest for UPR/HF/MC and UPR/HFC/MC composites and was improved by 112% and 58%, respectively.

Despite the LOI value for UPR/hemp fiber (HF) and UPR/HFP composites not changing much, it increased for UPR/HFP/MC composites from 19.3% to 26.3% compared to pure UPR.

The addition of HP or HFP to UPR only slightly improved the PHRR and THR. With the presence of MC, these features were decreased by 54% and 49%, respectively, for the UPR/HFP composites.

UP/HF and UP/HFP composites exhibited higher TSR than pure UPR, whereas UP/HFP/MC composite decreased its value from 4974.4 m^2^/m^2^ to 3517.8 m^2^/m^2^.

Overview of additive flame retardants for thermosetting resins are shown in Table 3.

## 6. Conclusions

Thermosetting resins pose a significant fire hazard. Therefore, their high flammability has to be reduced in order to obtain an adequate level of fire protection. The diversity of FRs enables their use as a polymer matrix of composites in different industrial fields such as the electrical industry or the commercial and military aircraft industries.

Although traditional FRs are commonly applied, there is a growing interest in bio-based FRs, which are a particular subject of EP research at this moment. In this review article, various FRs and their modes of action were considered in the fire-retardant application of thermosetting resins.

The initial temperature of composites containing bio-based flame retardants is mostly lower than for pure resin. It has been seen that flame inhibition strategies are mostly based on the forming of protective thermal barrier layers, and thus the char residue is significantly increased. However, capturing free radicals by some derivatives of itaconic acid and lychee peel, as well as diluting combustible compounds by some derivatives of alginate, phytic acid, and amino acid, is also observed.

In some cases, although clearly effective, FRs considered for the improvement of fire performance of thermosetting resins may adversely affect mechanical properties. In addition, there is a lack of efficient, high throughput technologies for obtaining composites with bio-based FRs. As can be seen from the bio-based FRs investigated above, the highest amount of additive is needed for FRs made from biowaste and FRs based on aminoacid. Such a serious amount of additives may have an adverse effect on their properties. However, an evaluation of the mechanical performance of composites is not always included in flammability studies. It is a great challenge to sustain good mechanical properties of composites in line with the improvement of flame retardancy, and this issue needs to be solved in the near future.

## Figures and Tables

**Table 1:** Major biomass components [11].

Group of Compounds	Subgroup	Example	Molecular Formula
Carbohydrates	Monosaccharides	Glucose	C_6_H_12_O_6_
	Polysaccharides	Starch	(C_6_H_10_O_5_)n
		Cellulose	(C_6_H_10_O_5_)n
		Xylose	C_5_H_10_O_5_
Phenolic compounds	Lignin	Coniferyl, coumaryl, and sinapyl alcohols	C_9_H_10_O_2_, C_10_H_12_O_3_, C_11_H_14_O_4_
Oils	Triglycerides	Oleic acid	C_18_H_34_O_2_
Proteins	Amino acids	Alanine	C_3_H_7_NO_2_

**Table 2:** Chemical composition of dry plant biomass and dry waste biomass [15].

Biomass	Cellulose [wt%]	Hemicelluloses [wt%]	Lignin [%]
White cotton	94–96	1–2	<1
Brown cotton	85–88	2–3	5–7
Flax	85–88	5–6	3–5
Softwood	46–48	20–23	27–28
Hardwood	44–46	25–27	22–25
Bagasse	37–39	23–25	19–21
Corn stalks	35–37	24–26	18–20
Corn cobs	34–36	36–38	9–11
Corn stover	35–37	28–30	18–20
Wheat straw	34–36	28–30	15–17
Rice straw	34–36	25–27	7–9
Switchgrass	36–38	26–28	17–19
Waste of textile	97–98	1–2	<1
Cotton linter	95–96	1–2	<1
Used office paper	60–62	4–6	1–2
Used newspaper	38–40	18–20	20–22
Used cardboard	58–60	14–15	10–12
Olive pomace	23–25	22–24	32–34

**Table 3:** Overview of additive flame retardants for thermosetting resins.

Flame Retardant	Type of Compound	Mode of Action	Effects	The Optimum Amount of Additive	Reference
**Flame retardants for epoxy resin**					
Core-shell graphitic carbon nitride/zinc phytate (PIPT)	Derivative of phytic acid	Forming stable, dense char layer and limiting heat transfer by labyrinth effect	Increase LOI Reduce PHRR rate and THR Prolong TTI Reduce FGI Reduce PSPR and TSP Reduce effective heat of combustion Increase residue mass Reduce tensile strength, elongation at , and impact strength	5 phr	[75]
Intumescent flame retardant system (APP/PER/MEL/eggshell)	Biowaste	Forming thermally stable char layer blocking mass and heat transfer between gas and condensed phases	Increase LOI Reduce PHR and THR Reduce TSR Reduce smoke density rating Increase residue mass	40 wt% (wt% of eggshell)	[76]
Cobalt alginate	Derivative of alginate	Forming a thin char layer and diluting combustible gases	Increase LOI Remain unchanged TTI Reduce PHRR Slightly increase THR Reduce FIGRA Reduce TSP Reduce the smoke density Increase char residue Reduce tensile strength, tensile modulus, and flexural strength are lower Improve the impact strength	3 wt%	[77]
Piperazine phytate	Derivative of phytic acid	Degradation of ammonium compounds resulting in NH_3_ release, diluting flammable gases, and promoting char formation	Increase LOI Reduce PHRR and THR Reduce PSPR and TSP Increase residue mass Reduce FIGRA Reduce tensile strength and elongation at	15 wt%	[78]
Fly ash (FA)/modified with HCl/modified with NaOH	Biowaste	Not mentioned	Decrease burning rate Increase LOI Insignificantly reduces flexural, tensile, and impact strength Increase compressive strength	20 wt%	[79]
ITA-HBP	Derivative of itaconic acid	Forming of the char layer, blocking heat and oxygen permeation. Releasing free radicals promotes pyrolysis. Releasing volatile compounds diluting flammable gases.	Increase LOI Make EP self-extinguishing Reduce PHRR and THR Shorten TTI Reduce TSR Improve impact and flexural strength, and toughness	20 phr	[80]
Lychee peel	Biowaste	Forming of the rich-carbon char layer, blocking fuel transport. Absorbing free radicals.	Slightly increase LOI Decrease the burning rate Increase tensile strength, compressive strength, and impact strength Decrease flexural strength	20 wt%	[81]
Gallic acid, Ellagic acid, Derivatives of acids (GA, EA, GAD, EAD)	Derivatives of gallic and ellagic acids	Forming of the protective layer slowing pyrolysis.	Shorten TTI Reduce PHRR and THR Reduce burning rate	10 wt%	[41]
**Flame retardants for unsaturated polyester resin**					
L-histidinium dihydrogen phosphate–phosphoric acid (LHP)	Derivative of aminoacid	Forming of the char layer blocks heat transport and slows the burning rate. Probably also a heat sink effect and diluting oxygen concentration due to decomposition of propionic acid.	Makes UPR self-extinguishing Reduces PHRR and THR Reduces TSR Reduces smoke density	30 wt%	[86]
Phosphorylated chitosan-coated carbon microspheres (PCCCM	Derivative of chitosan	Forming of the tortuous char layer blocks heat and pyrolysis products transport. Dilution of combustible gases.	Slightly increases LOI value Increases residue mass Shortens TTI Reduces PHRR Slightly increases THR Reduces PSPR and TSP	3 wt%	[87]
LbL assembled diatomite based on chitosan and APP (DCHAPP)	Derivative of chitosan	Forming of compact and colloidal char layer blocking heat transport and preventing the transmission of pyrolysis compounds.	Increases LOI Increases char residue Reduces PHRR and THR Prolongs TTI Reduces SPR and TSR Slightly improves flexural and tensile strength	25 phr (9 bilayers)	[88]
PN-lignin	Derivative of lignin	Forming of the char layer blocks heat and gas transfer.	Decreases the burning rate Slightly improves thermal stability	12,5 wt%	[89]
Modified hemp fiber combined with melamine cyanurate	Biowaste	Forming of the expandable char layer blocks heat transfer and isolation from the air.	Increase LOI Decreases PHRR and THR Decreases TSR Increases residue mass	30 wt% (hemp fiber 3 wt%)	[90]

## Data Availability

Not applicable.
